# The role of 3D printed models in the teaching of human anatomy: a systematic review and meta-analysis

**DOI:** 10.1186/s12909-020-02242-x

**Published:** 2020-09-29

**Authors:** Zhen Ye, Aishe Dun, Hanming Jiang, Cuifang Nie, Shulian Zhao, Tao Wang, Jing Zhai

**Affiliations:** 1 Department of Molecular Biology, Basic Medical College, Shandong First Medical University & Shandong Academy of Medical Sciences, Tai’an, Shandong P.R. China; 2 Department of Anatomy, Basic Medical College, Shandong First Medical University & Shandong Academy of Medical Sciences, Tai’an, Shandong P.R. China; 3 Department of Infectious Disease, Tai’an Central Hospital, Tai’an, Shandong P.R. China

**Keywords:** Three-dimensional printing, Medical education, Anatomy

## Abstract

**Background:**

Three-dimensional (3D) printing is an emerging technology widely used in medical education. However, its role in the teaching of human anatomy needs further evaluation.

**Methods:**

PubMed, Embase, EBSCO, SpringerLink, and Nature databases were searched systematically for studies published from January 2011 to April 2020 in the English language. GRADEprofiler software was used to evaluate the quality of literature. In this study, a meta-analysis of continuous and binary data was conducted. Both descriptive and statistical analyses were used.

**Results:**

Comparing the post-training tests in neuroanatomy, cardiac anatomy, and abdominal anatomy, the standardized mean difference (SMD) of the 3D group and the conventional group were 1.27, 0.37, and 2.01, respectively (*p* < 0.05). For 3D vs. cadaver and 3D vs. 2D, the SMD were 0.69 and 1.05, respectively (*p <* 0.05). For answering time, the SMD of the 3D group vs. conventional group was – 0.61 (*P* < 0.05). For 3D print usefulness, RR = 2.29(*P <* 0.05). Five of the six studies showed that satisfaction of the 3D group was higher than that of the conventional group. Two studies showed that accuracy of answering questions in the 3D group was higher than that in the conventional group.

**Conclusions:**

Compared with students in the conventional group, those in the 3D printing group had advantages in accuracy and answering time. In the test of anatomical knowledge, the test results of students in the 3D group were not inferior (higher or equal) to those in the conventional group. The post-training test results of the 3D group were higher than those in the cadaver or 2D group. More students in the 3D printing group were satisfied with their learning compared with the conventional group. The results could be influenced by the quality of the randomized controlled trials. In a framework of ethical rigor, the application of the 3D printing model in human anatomy teaching is expected to grow further.

## Background

Three-dimensional (3D) printing (also known as additive manufacturing) is a process in which a 3D computer model is transformed into a physical object [1]. Through computer control, the “printed materials” are stacked layer by layer, until the physical object matches the blueprint on the computer. Commonly used materials for 3D printing include durable nylon, gypsum, aluminum, textile materials, and polylactic acid [2, 3]. The future of 3D printing seems to involve printing more realistic models using different materials [4]. Compared with other tissue engineering scaffolds and rapid prototyping technology, 3D printing has the following advantages: high accuracy, good integration, fast reconstruction, and low cost [5]. In addition, 3D printing models can make it easy for people to understand complex physical structures and print models that are difficult to obtain [6].

Three-dimensional printing has a wide range of applications, including applications in space science, technology, and medicine. For example, technology can be used to scan the human body with magnetic resonance imaging and a computerized tomography scan. It can then replicate human structures with multiple layers of resin [7]. Resin is laid in layers that finally generates solid models. It is a potentially disruptive technology that can improve surgical education and clinical practice [5]. For example, 3D printing of cerebral arteriovenous malformation models is helpful for preoperative patient consultation, surgical planning, and training [8]. These models can explain the patient’s illness, improve the doctor-patient relationship, and increase the patient’s confidence in the treatment process. They also provide patients with auxiliary education and inform them about normal and abnormal body structures, which is conducive to improving doctor-patient relationships [8]. Diment et al. [9] used a descriptive-analytical method to analyze the application of 3D printing models in clinical fields and proposed that 3D models have effective applications. Bai et al. [10] reported in their meta-analysis that 3D print-assisted surgery was better than conventional surgery in terms of operation time, blood loss, and favorable outcomes. Compared with a conventional group, the 3D printing group showed a shorter operation time, less intraoperative blood loss, and faster healing time in patients with tibial plateau fractures, suggesting that 3D printing technology-based treatment was appropriate for tibial plateau fractures [11]. Langridge et al. [12] used descriptive statistical methods to report the role of 3D printed models in surgical education. The author concluded that 3D printing technology has a wide range of potential applications in surgical education and training.

Three-dimensional printing is used to train residents for anatomy education. This technology has shown great potential as an educational tool in areas such as autopsy, plasticization, computer simulation, and anatomical modeling and images [13]. In recent decades, 3D printing has been employed in the teaching of anatomy to medical students [13]. It is feasible to use this technology to produce high-fidelity models of heart abnormalities. These models impart knowledge about the heart to students and augment their interest in learning [14]. They can also be replicated in large numbers, providing more models for students to use for learning and practicing their skills. One study reported that students found 3D printed models more flexible and durable compared to conventional plastic models [8]. In addition, 3D printing has relatively low production costs, generates an accurate anatomical structure, and demonstrates normal or pathological structural changes [15, 16]. Conventional cadaver model anatomy training presents several difficulties including the cost of the cadaver, ethical issues, and the application of formalin preservatives.

The applications of 3D printed models have been investigated in many meta-analyses [9–11, 17], largely in the field of surgery. Our study evaluated the application of 3D printed models in medical education. Meta-analysis in medical education is rare. Our research comprised the following processes: (1) A wide range of source data, comprising categorical and continuous variables, were analyzed; (2) meta-statistical analysis and descriptive analysis were performed; (3) a merger analysis of the effects was performed after deleting individual studies, and the data were visualized; and (4) some studies did not provide the standard deviation (SD) [18, 19], and hence, we estimated it through a formula.

In this study, we compared 3D printed models with conventional models to understand the advantages and disadvantages of 3D printed models, and to provide a better understanding of their use in the teaching of anatomy. In our research, conventional teaching models of anatomy include cadavers, plastic products, and two-dimensional (2D) anatomical pictures.

## Methods

This study complies with the Preferred Reporting Items for Systematic Reviews and Meta-Analyses (PRISMA) guidelines [20].

### Study identification and eligibility criteria

We systematically searched PubMed, Embase, EBSCO, SpringerLink, and Nature databases using the following search terms: (“anatomy education” OR “anatomy teaching”) and (“3D printing” OR “three-dimensional printing” OR “3D printed”) and (“student” OR “resident”). We included studies in the English language published from January 2011 to April 2020. To avoid omitting literature, we selected one or two of the key words in the above parentheses, matched them randomly, and retrieved the literature again. Next, we read the titles and abstracts online. If the description of the abstract did not match the target literature it was excluded; if the description of the abstract was generally in line with our purpose, the full text was downloaded and further screened. If the full text could not be downloaded, we emailed the respective authors for the articles. A study was eligible in the meta-analysis if: (1) the anatomy or structure of the human body was identified, (2) a normal or diseased condition was mentioned, (3) a randomized controlled study was conducted, (4) teaching for medical students or junior residents was involved, (5) there were at least 10 participants in the experimental group and the control group, and (6) there were clear experimental indicators and experimental data. We excluded studies that (1) had no control groups; (2) used animal models; (3) were case reports, letters, comments, reviews, or other meta-analyses; (4) did not allow extraction of the required data; (5) included republished data; (6) patient education; (7) surgical technical training; (8) 3D computer models; or (9) were unsuitable for other reasons.

### Data extraction

For each study, two reviewers (J.H, N.C) independently extracted the first author, publication year, country, the number of experimental and control groups, and a specific comparison between the two groups. Disagreements were resolved through discussion.

### Literature quality assessment

We used the GRADEprofiler software (Version 3.6, GRADE Working Group) to evaluate the quality of literature included in the study.

The assessment methods [21] included the following items: (1) Experimental design, (2) Risk of bias, (3) Inconsistency, (4) Indirectness, (5) Imprecision, and (6) Publication bias. The quality was assessed by two independent reviewers (J.H, Y.Z). If there is disagreement among the staff, it was settled through consultation.

The results of the quality evaluation are divided into four levels, defined as follows.

High quality: Further research is very unlikely to change our confidence in the estimate of effect.

Moderate quality: Further research is likely to have an important impact on our confidence in the estimate of effect and may change the estimate.

Low quality: Further research is very likely to have an important impact on our confidence in the estimate of effect and is likely to change the estimate.

Very low quality: We are very uncertain about the estimate.

If the quality of literature is evaluated as “High” or “Moderate,” we assumed that the result will be reliable. If the quality evaluation of the literature is “Low,” we assumed that the results are less reliable.

### Statistical analysis

This study included a meta-analysis of categorical and continuous variables. For the categorical data, we used R version 3.5 (http://www.r-project.org/) to calculate relative risk (RR) values and to compare the results. For continuous data, due to different scoring standards, a standardized mean difference (SMD) was used to compare the results. For the conjoint analysis of continuous variables, we needed to know the mean ($\overline{\boldsymbol{X}}$) and SD of the experimental and control groups. The specific $\overline{\boldsymbol{X}}$ and SD values were not provided in the two papers [18, 19]. Hence, we calculated them using published formulas as follows [22].

$$ \overline{X}\approx \left\{\begin{array}{c}\frac{\mathrm{Q}1+\mathrm{Q}3+2\mathrm{m}}{4}\\ {}m,\kern0.5em n>25\end{array},\kern0.5em n\kern0.5em \leq \kern0.5em 25\right\} $$

$$ \mathrm{SD}\approx \left\{\begin{array}{c}\frac{1}{\sqrt{12}}\ast {\left[{\left(\mathrm{Q}3-\mathrm{Q}1\right)}^2+\frac{{\left(\mathrm{Q}1+\mathrm{Q}3-2\mathrm{m}\right)}^2}{4}\right]}^{\frac{1}{2}},\mathrm{n}\le 15\\ {}\frac{\mathrm{Q}3-\mathrm{Q}1}{4},15<\mathrm{n}\le 70\\ {}\frac{\mathrm{Q}3-\mathrm{Q}1}{6},\mathrm{n}>70\end{array}\right. $$

We used the random effects model to merge the data. The funnel chart was examined using Egger’s and Begg’s tests [23]. It indicated that there was a publication bias of *p* < 0.05. Data stability was assessed through a sensitivity analysis. We performed this analysis by deleting a study, combining effects, and comparing the results of a sufficient number of studies (3 < *n* < 10). In the above study, we used the R language by loading “meta” packages (https://cran.r-project.org/web/packages/meta/index.html).

## Results

### Characteristics of the eligible studies

We searched the relevant databases and read the abstracts and full texts of articles found during this search. Seventeen studies were included in the analysis [13, 18, 19, 24–36].

The publication period of the retrieved literature was between 2015 and 2020. Nine of the seventeen studies were from China, four from the United States, and one each from the United Kingdom, Australia, Japan, and Singapore, respectively. Six studies investigated the use of the models of the nervous system, while five investigated the use of heart models (Table 1). The quality evaluation of most literature studies was high or moderate. Details on the literature quality assessment of the included studies appear in Supplementary Table S1. In all the 17 studies, subjects were divided into groups by randomized controlled grouping. In a few studies, the method of generating random numbers was described in detail. In none of them was the use of any blind method described. Since the number of students included in these studies was relatively small, there may have been some bias [18, 25, 28].

**Table 1:** **Basic** characteristics of the 17 included studies

Study	Year	Region	3D vs. conventional	Organ	Observe
Chen	2020	China	23 vs. 24 (2D images)	Gastrocolic Trunk	Test results, satisfaction
Tanner	2020	United States	45 vs. 43 (cadaver materials)	Skull	Test results, satisfaction
Yi	2019	China	20 vs. 20 (2D images)	Head	Test results
Bangeas	2019	United States	10 vs. 10 (2D images)	Colon, rectum	Satisfaction, usefulness, choice tendency, test results
Hojo	2019	Japan	51 vs. 51 (textbook group/2D images)	Pelvis	Test results
Cai	2018	Singapore	17 vs. 18 (2D images)	Knee joint	Accuracy
Huang	2018	China	47 vs. 47 (physical model)	Acetabulum	Objective tests, usefulness, accuracy, choice tendency
Lin	2018	China	22 vs. 20 (atlas)	Head	Test results
Su	2018	China	32 vs. 31 (CT)	Heart	Test results
Wu	2018	China	45 vs. 45 (CT)	Spine, pelvis, upper limb,	Satisfaction, answering time, test results
				lower limb	
Chen	2017	China	26 vs. 27 (cadaver materials)	Skull	Test results
Jones	2017	United States	17 vs. 19(2D images)	Vascular rings and slings	Test results
Loke	2017	United States	18 vs. 17 (2D images)	Anatomy of congenital	Knowledge acquisition, satisfaction, test results
				heart disease	
Smith	2017	United Kingdom	66 vs. 61 (cadaver materials)	Heart, lung	Test results
Wang	2017	China	17 vs. 17 (plastic cardiac model)	Heart	Satisfaction, answering time, choice tendency
Lim	2016	Australia	16 vs. 18 (cadaveric materials)	Heart	Test results
Li	2015	China	21 vs. 22 (female, CT);	Spine	Usefulness, answering time
			19 vs. 18 (male, CT)		

### Post-training tests

#### Nervous system model

**Fig. 1:**
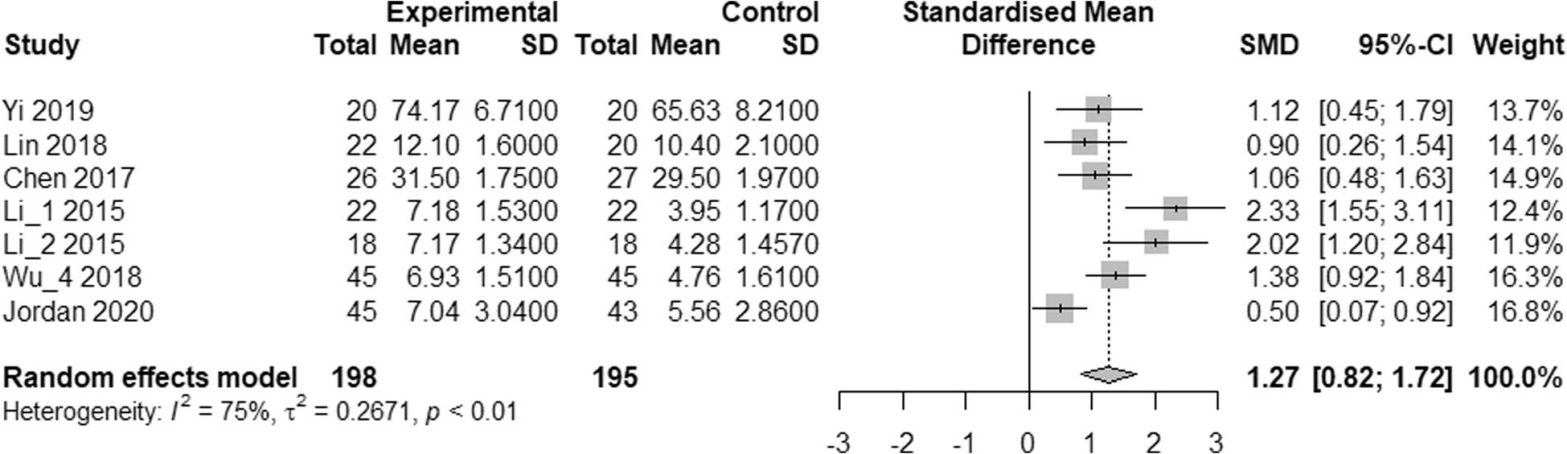
Comparison of test results of the experimental and the control groups for nervous system models. A meta-analysis of continuous data

Six studies compared 3D printed models with conventional nervous system models [17, 19, 24, 31–33]. There were 198 in the experimental group and 195 in the control group. The results showed a significant difference between the two groups (SMD: 1.27, 95% confidence interval [CI]: 0.82–1.72, *P* < 0.001; Fig. 1). This showed that the post-training test scores of the 3D group were higher than the conventional group.

#### Heart model

Five studies compared 3D printed heart models with conventional heart models [25–28, 34]. These studies included a total of 100 participants in the 3D printing group and 102 participants in the conventional group. Tests were administered after the instructions for using the models or conventional methods had been given. The test score variables were continuous. Due to the different test score standards used in different studies, we used an SMD to merge the means. The results showed no significant difference between the two groups (SMD: 0.37, 95% confidence interval [CI]: – 0.25–0.98, *P* = 0.24; Fig. S1). Therefore, the post-training test scores of the 3D group were not higher than those of the conventional group.

#### Abdominal anatomy

Three studies compared 3D printed models with conventional abdominal anatomy models [17, 18, 32]. The results showed that there was a significant difference between the two groups (SMD: 2.01, 95% confidence interval [CI]: 0.55–3.46, *P* = 0.007; Fig. S2). Therefore, the test results of the 3D group were higher than that of the control group.

##### D vs. cadaver

**Fig. 2:**
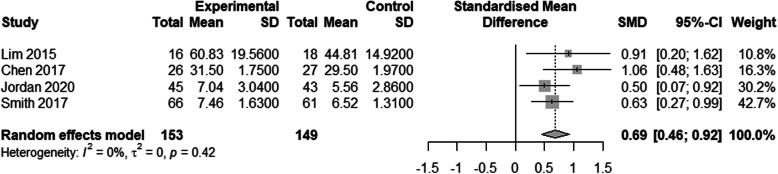
Comparison of test results of the 3D and cadaver groups. A meta-analysis of continuous data

Four studies compared 3D printed models with cadaver specimens [19, 25, 30, 33]. There were 153 in the experimental group and 149 in the cadaver specimen group. The results showed a significant difference between the two groups (SMD: 0.69, 95% confidence interval [CI]: 0.46-0.92, *P* < 0.001; Fig. 2). The test results of the 3D group were higher than that of the cadaver specimen group.

##### D vs. 2D

Ten studies compared 3D printed models with 2D pictures [18, 24, 26, 27, 29, 31, 32, 34–36]. There were 379 3D printed models in the experimental group and 378 2D pictures in the control group. The results showed a significant difference between the two groups (SMD: 1.05, 95% confidence interval [CI]: 0.64–1.64, *P* < 0.001; Fig. S3). The test results of the 3D group were higher than that of the 2D group.

### Answering time

Three studies compared the difference in answering times between the 3D printing groups and conventional groups [24, 28, 29]. The random effects model suggested a statistical significance (SMD: – 0.61, 95% CI: – 0.98 to – 0.24, *P* = 0.001; Fig. S4). This also suggested that the answering time in the 3D printing groups was shorter compared to the conventional groups.

### Usefulness

**Fig. 3:**
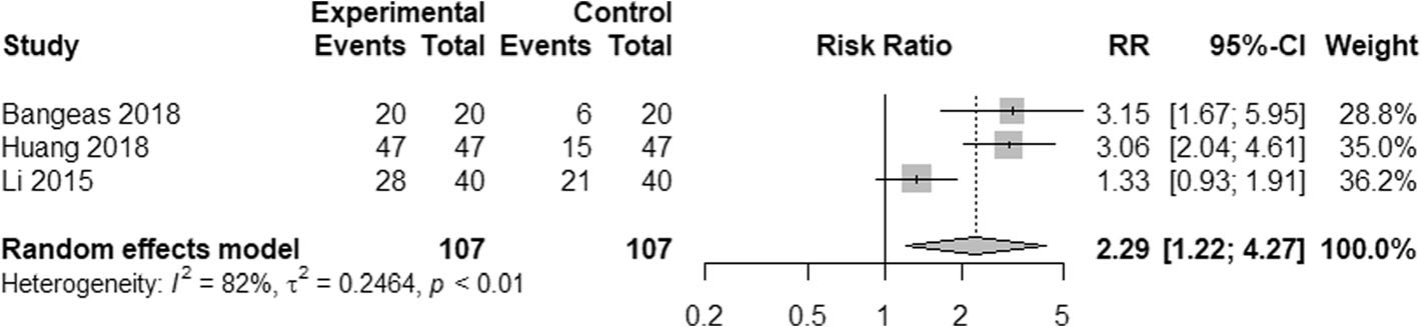
Compared 3D printed models with conventional models concerning utility. A meta-analysis of binary data

Three studies compared 3D printed models to conventional models regarding utility [18, 37, 38]. The random effects models suggested a statistical significance (RR = 2.29, 95% CI: 1.22–4.27, *P* = 0.008, Fig. 3). This suggested that the instruction for 3D printing was more useful compared to the instruction for conventional models.

### Satisfaction

Six studies described the level of satisfaction in the 3D printing and conventional groups [13, 18, 26, 28, 33, 36]. Results from five studies indicated that students in the 3D printing group were more satisfied compared to students in the conventional group. Only one article reported that there was no statistical difference in satisfaction between the students in the 3D printing group and those in the conventional group (Table S2).

### Accuracy

Two studies investigated the answering accuracy in the 3D printing and conventional groups [39, 40]. The two studies are descriptive and do not incorporate data. These studies found that answering accuracy in the 3D printing group was higher compared to the conventional group (Table S3).

### Sensitivity analysis

Regarding studies about the nervous system, each time a study was deleted and the rest of the data were combined, the *P*-values were less than 0.05 (Fig. S5), which suggested that the result was stable and reliable. Similarly, while comparing 3D models with cadavers, we omitted one study at a time, and the pooled estimates were calculated in both the 3D printing and conventional groups (Fig. S6). Each time a study was ignored, the pooled estimates were found to be < 0.05, which suggested that the result was stable and reliable as well.

### Test for publication bias

In the funnel plots of the 3D printing model and the conventional model of performance testing (nervous system, Fig. S7), both Egger’s and Begg’s tests showed a *P*-value of > 0.05, indicating an even and symmetrical distribution with no publication bias. However, the integrated study of 3D vs. 2D showed a *P-*value of < 0.05, suggesting that there may be publication bias.

## Discussion

3D printing has become more popular in medical education in recent years. The 17 studies included in this analysis were published between 2015 and 2020 (Table 1). The results showed that the 3D group was superior to the control group in terms of test scores, accuracy, and student satisfaction, when the literature quality was assessed at a high or moderate quality level (Table S1). The test results of the 3D group were higher than those of the cadaver group and 2D group, respectively. In the nervous system model and 3D vs. 2D, sensitivity analysis suggested that the results were reliable and stable as well. The results showed that the heart model test scores of the 3D group was not higher than that of the control group. The literature quality for this was evaluated as low (Table S1). This suggests that in the heart model, the comparison between the two groups may be less stable. The 3D printing group was better than the control group in usefulness and test time.

In our study, students in the 3D printing groups took less time to answer questions compared to the conventional groups. Wu et al. [29] reported that, compared with a conventional group, students in the 3D printing group spent less time answering questions on the pelvis and spine. However, there was no significant difference in the time spent on answering questions related to the upper and lower limbs between the groups. Li et al. [37] reported that both male and female students spent less time answering questions on the spine models in the 3D group as compared with a conventional group. The different results of the above research may be due to the variations in students and organs. In general, 3D printing groups took less time to answer questions compared to the conventional groups. However, the quality evaluation of the literature is low for this, indicating that the result may not be very stable (Table S1). Three studies compared 3D printed models with conventional models regarding utility [18, 37, 40], and the random effects model suggested a statistical significance. In terms of 3D print usefulness, 3D printed models were found to be more useful compared with conventional models. However, the quality evaluation of the literature for this is low as well (Table S1). Six studies investigated the satisfaction of students in the 3D printing and conventional groups with their learning. Five of these studies showed that students in the 3D group were more satisfied than the conventional group. Only one article mentioned that there was no statistical difference between the two groups. These results indicate that there was more satisfaction among students in the 3D printing groups than among students in the conventional groups (Table S2). Three-dimensional printing is embraced by students and shows the innovation of new, exciting technology. Two studies have investigated the accuracy in answering questions among students in 3D printing groups and conventional groups [39, 40]. Students in the 3D printing groups showed more accuracy in answering questions compared with students in the conventional groups (Supplementary Table). Similar to the post-training test, high accuracy in answering questions represents high test scores.

The visual funnel diagram was tested for symmetry and was found to be symmetrical (Fig. S6). By loading the “meta” package, both Egger’s and Begg’s tests showed a *P*-value of > 0.05, indicating the absence of a publication bias.

In the past, for a medical student, the primary learning object was often a real human body. Some of the surgical teaching and research departments in hospitals have anatomical maps displayed to help students learn. Today, some departments teach students about human anatomy through 3D computer graphics. However, 3D printing has the advantages of high accuracy, good integration, fast reconstruction, and low cost. Technology has gradually entered the medical classroom. Using 3D printing technology to create an anatomical customized model to fully understand the anatomical relationship between lesions and complex surrounding structures will be very useful for practical and teaching purposes [37].

Although the field is still relatively new, some studies have shown that education that employs 3D printing can replace or supplement conventional education [12].

Three-dimensional printed models also have some shortcomings. If students only have access to “scaled” models, it could lead to a lack of understanding of real size and relation to other anatomical components [38]. The accuracy of 3D printed models remains a challenge and they have yet to completely replace human structures [41]. The costs associated with various materials and equipment are also a problem. Moreover, the ethical issues regarding 3D printed models should not be ignored. The research on 3D printing of the foregut, organ, and archived fetal materials using a donated body or 3D files on the Internet, is of ethical significance [42]. Without the permission of the donor, 3D printing of the body may lead to a lack of “reasonable” informed consent, which is ethically questionable. At worst, if the models are sold for a profit, it could be interpreted as illegal [43]. However, despite potential cost constraints, the prices of 3D printing equipment, materials, and software have been declining [40, 44], and more educational 3D printing models are becoming learning tools for students [12]. People donated their bodies for anatomical studies as an altruistic act for the benefit of medical education. Because such a valuable resource is both scarce and costly, 3D printing technology can duplicate the anatomical parts of the human body at a low cost, providing useful anatomical resources for medical education in poor areas. Hence, we must take heed of the ethical considerations and abide by them when using 3D printed anatomical models. However, with good ethical rigor, we hope that 3D printing models can not only play a role in surgery and communication, but also in anatomy classes. In the future, 3D printing would revolutionize anatomy when poly-material printing is perfected [45].

## Limitations

It is necessary to acknowledge the limitations of this study. Most of the papers did not specifically describe the procedures of randomization, such as the method of generating random numbers. None of the research indicated whether they were blind. Furthermore, most of the studies were heterogeneous. There are possible reasons for heterogeneity, such as the difference in the overall quality of students in different countries, the quality of teachers, the contents and objectives of teaching, and the contents of questionnaires. Additionally, the sample sizes in most of the studies were small.

## Conclusions

In teaching the human body using 3D printed models, the test results will not be inferior to that of the conventional teaching group. Compared with the cadaver or 2D group, the 3D group had higher test scores. Compared with the conventional group, students in the 3D group had higher test accuracy, and the students found the 3D model more useful. Most of the students in the 3D printing group were more satisfied with their learning than those in the conventional group. The confidence in these results may be affected by factors such as the poor design quality in some of the randomized controlled trials, the difficulty of the test papers, or the background knowledge of students and teachers. In general, in a good ethical situation, the application of a 3D printing model in human anatomy is worthy of exploration and adaption.

## Supplementary information

**Additional file 1.**

## Data Availability

All raw data used in this systematic review and meta-analysis were available from published articles.

## Abbreviations

3D: Three-dimensional
SD: Standard deviation
RR: Relative risk
SMD: Standardized mean difference
