# Supplementary material for: The role of 3D printed models in the teaching of human anatomy: a systematic review and meta-analysis
Source: BMC Med Educ. 2020 Sep 29;20:335. doi: 10.1186/s12909-020-02242-x (PMC7523371; doi:10.1186/s12909-020-02242-x)
Supplement: Supplementary file 1 — Additional file 1. [file 12909_2020_2242_MOESM1_ESM.docx]

**Supplementary materials for “The role of 3D printed models in the teaching of human anatomy: a systematic review and meta-analysis”**

Zhen Ye, Aishe Dun, Hanming Jiang, Cuifang Nie, Shulian Zhao, Tao Wang, Jing Zhai

**Table S1** Literature quality assessment included in the analysis.

| Quality assessment | | | | | | | No of patients | | Quality | Importance |
| --- | --- | --- | --- | --- | --- | --- | --- | --- | --- | --- |
|  |  |  |  |  |  |  |  |  |  |  |
| No of studies | Design | Risk of bias | Inconsistency | Indirectness | Imprecision | Other considerations | Conventional group | 3D printing group |  |  |
| Post-training tests_ Nervous system model_3D vs. control | | | | | | | | | | |
| 6 | Randomized trials | Serious^1^ | No serious inconsistency | No serious indirectness | No serious imprecision | Undetected^3^  Strong association^2^ | 152 | 153 | HIGH | CRITICAL |
| Post-training tests_ Heart model _3D vs. control | | | | | | | | | | |
| 5 | Randomized trials | Serious^1^ | Serious | No serious indirectness | No serious imprecision | NA  Strong association^2^ | 83 | 83 | LOW | Not IMPORTANT |
| Post-training tests_ Abdominal anatomy_3D vs. control | | | | | | | | | | |
| 3 | Randomized trials | Serious^1^ | No serious inconsistency | No serious indirectness | No serious imprecision | NA | 72 | 72 | MODERATE | IMPORTANT |
| Post-training tests_3D vs. Cadaver | | | | | | | | | | |
| 4 | Randomized trials | Serious^1^ | No serious inconsistency | No serious indirectness | No serious imprecision | NA | 153 | 149 | MODERATE | IMPORTANT |
| Post-training tests_3D vs. 2D | | | | | | | | | | |
| 10 | Randomized trials | Serious^1^ | No serious inconsistency | No serious indirectness | No serious imprecision | Reporting bias^3^  Strong association^2^ | 379 | 378 | MODERATE | IMPORTANT |
| Answering time_3D vs. control | | | | | | | | | | |
| 3 | Randomized trials | Serious^1^ | Serious | No serious indirectness | No serious imprecision | NA | 237 | 237 | LOW | Not IMPORTANT |
| Usefulness_3D vs. control | | | | | | | | | | |
| 3 | Randomized trials | Serious^1^ | Serious | No serious indirectness | No serious imprecision | NA | 95 | 107 | LOW | Not IMPORTANT |
| Satisfaction_3D vs. control | | | | | | | | | | |
| 6 | Randomized trials | Serious^1^ | Serious | No serious indirectness | No serious imprecision | NA  Strong association^2^ | 157 | 157 | MODERATE | IMPORTANT |
| Accuracy_3D vs. control | | | | | | | | | | |
| 2 | Randomized trials | Serious^1^ | No serious inconsistency | No serious indirectness | No serious imprecision | NA | 64 | 65 | MODERATE | IMPORTANT |
|  |  |  |  |  |  |  |  |  |  |  |

^1^ The papers did not specifically describe the procedures of randomization.
^2^ The number of included studies is no less than 5.
^3^ Publication bias was evaluated if a sufficient number of studies (n >= 5) were included.


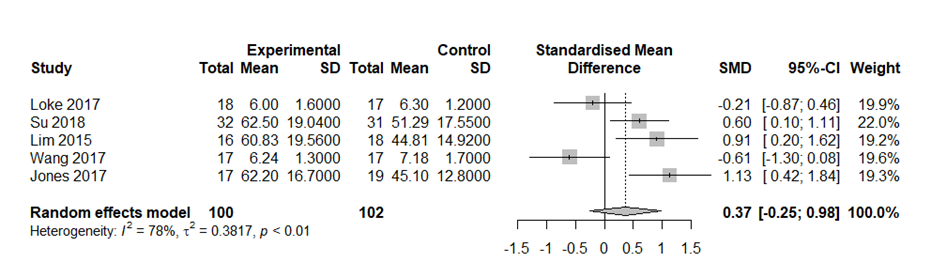


**Figure** **S1** Comparison of test results of the experimental and control groups for heart models.


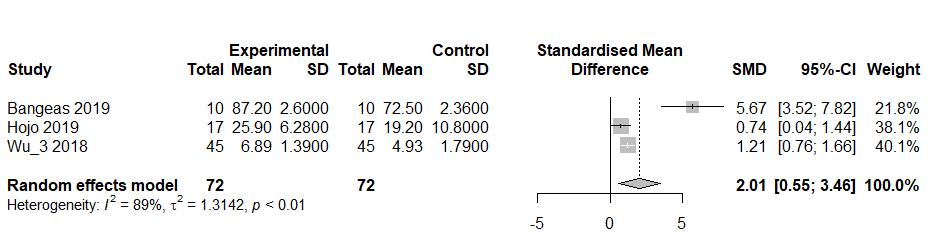


**Figure S2** Comparison of test results of the experimental and control groups for abdominal anatomy models.


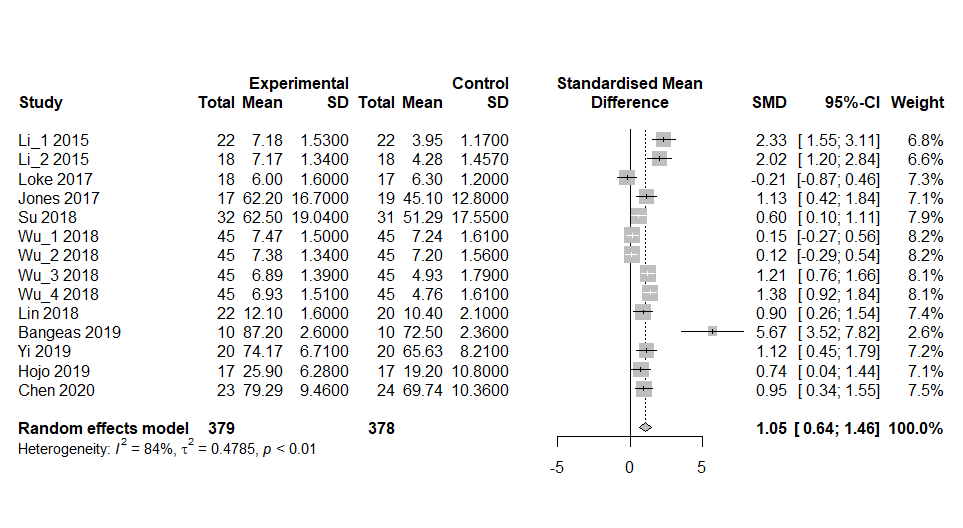


**Figure S3** Comparison of the test results in answering questions between the 3D and 2D groups.


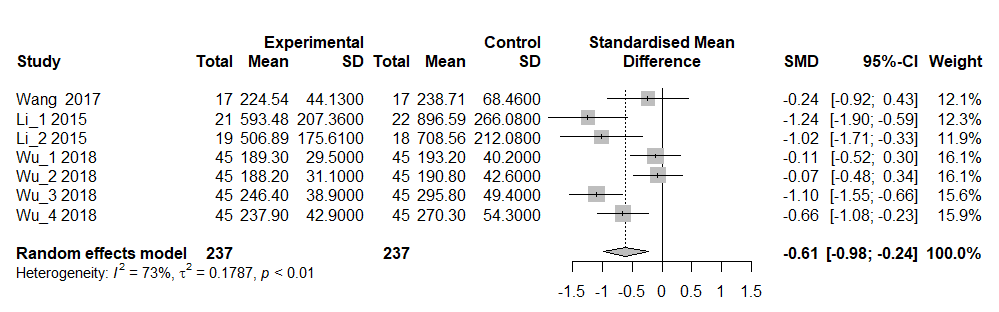


**Figure** **S4** Comparison of the time spent in answering questions between the experimental and control groups.


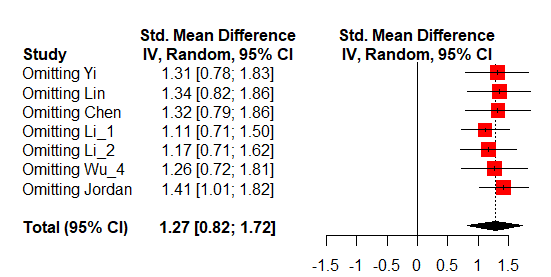


**Figure S5** Sensitivity analysis of the meta-analysis of test results for nervous system models in the experimental and control groups using the leave-one-out method. Li_1 and Li_2 were obtained from the same source literature. The data from Li_1 was obtained from the females, while that of Li_2 was obtained from the males. Wu_1 through Wu_4 were obtained from the same literature. Wu_1 data were obtained from the upper limbs, and Wu_2 data were obtained from the lower limbs. Wu_3 data was obtained from the pelvis, and Wu_4 data was obtained from the spine.


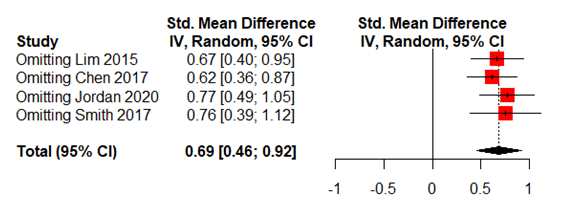


**Figure S6** Sensitivity analysis of the meta-analysis of test results in 3D and cadaver groups using the leave-one-out method.


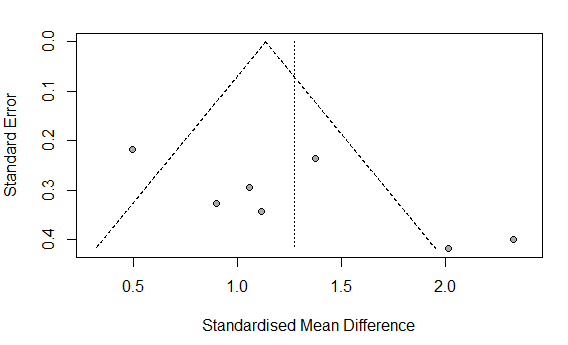


**Figure S7** Funnel plot of standardized mean difference of the test results for nervous system models in the experimental and control groups.

**Table S2** Six documents describing the satisfaction of 3D vs. conventional groups.

| Study | Description |  |
| --- | --- | --- |
| Bangeas, 2017 | Both groups were satisfied with the study about the use of 3D printing technology for educational purposes. |  |
| Loke, 2017 | The residents reported better satisfaction when using the 3D group as compared to the 2D group with an improvement in composite learner satisfaction score from 21 to 24. |  |
| Wang, 2017 | Compared with the Traditional Model Group, more students in the 3D Printing Group marked 9−10 points for satisfaction with teaching, although the difference was not significant. |  |
| Wu, 2018 | The mean score in the visual analog scale for satisfaction was 7.49 ± 1.38 in the 3D group and 5.80 ± 1.30 in the traditional group (P < 0.05). |  |
| Chen, 2020 | For general satisfaction, 21  interns who in the 3D group felt satisfied, were higher than those of the control  group (p = 0.032). |  |
| Tanner, 2020 | The 3D group had significantly higher Likert scores as compared to the control group (P < 0.05 for all questions). |  |

**Table S3** The two documents described the answering accuracy of 3D vs. conventional groups.

| Study | | Description | |  |
| --- | --- | --- | --- | --- |
| Cai, 2018 | | The students in the simulation learning group achieved a far better understanding than those in the traditional learning group, with mean values of 85.03 ± 10.13% and 70.71 ± 15.13%, respectively (P < 0.05). | |  |
| Huang, 2018 | | While testing major fracture lines and classification, the 3D group showed a clear advantage over the PM and VR groups. The rates of ‘all correct’ classifications were 27.7%, 27.7%, and 51.1% in the PM, VR, and 3D groups, respectively. | |  |
|  | |  |  |  |
